# The MELD-XI score predicts 3-year mortality in patients with chronic heart failure

**DOI:** 10.3389/fcvm.2022.985503

**Published:** 2022-08-11

**Authors:** Zebin Lin, Xia Liu, Li Xiao, Yin Li, Chenlu Qi, Siqi Song, Yiping Zhao, Lili Zou

**Affiliations:** ^1^Department of Geriatrics, Zhongshan Hospital Affiliated to Xiamen University, Xiamen, China; ^2^Department of Pulmonary and Critical Care Medicine, First Affiliated Hospital, Bengbu Medical College, Bengbu, China; ^3^Anhui Province Key Laboratory of Clinical and Preclinical Research in Respiratory Disease, Bengbu, China; ^4^Clinical Research Center for Respiratory Disease (tumor) in Anhui Province, Bengbu, China; ^5^Department of General Medicine, The Second Affiliated Hospital of Chongqing Medical University, Chongqing, China; ^6^Department of Hepatobiliary Surgery, The Second Affiliated Hospital of Chongqing Medical University, Chongqing, China; ^7^Department of Cardiology, The First Affiliated Hospital of University of Science and Technology of China, Hefei, China; ^8^Department of Cardiology, The Second Affiliated Hospital of Chongqing Medical University, Chongqing, China

**Keywords:** MELD-XI score, chronic heart failure, prediction model, creatinine, bilirubin

## Abstract

**Objectives:**

The relationship between the MELD-XI score, a modified version of the MELD score, and the long-term prognosis of hospitalized patients with chronic heart failure is unclear. The aim of this study was to determine the long-term prognostic relationship of MELD-XI score in patients with chronic heart failure.

**Methods:**

This is a retrospective cohort study of patients with chronic heart failure who were initially hospitalized in the Second Affiliated Hospital of Chongqing Medical University from February 2017 to December 2017. The primary clinical outcome was all-cause mortality within 3 years. Cox regression and lasso regression were used to screen variables and build a prognostic model. Combined with the MELD-XI score, the final model was adjusted, and the predictive ability of the model was evaluated. Survival curves were estimated using the Kaplan–Meier method and compared by the log rank test.

**Results:**

A total of 400 patients with chronic heart failure were included (median age 76 years, 51.5% female). During the 3-year follow-up period, there were 97 all-cause deaths, including 63 cardiac deaths. Six characteristic variables (NT-proBNP, BUN, RDW CV, Na^+^ and prealbumin) were selected by univariate Cox regression and lasso regression. Survival analysis results showed that elevated MELD-XI score at baseline predicted the risk of all-cause mortality at 3 years in patients (HR 3.19, 95% CI 2.11–4.82, *P* < 0.001; HR_adjusted_ 1.79, 95% CI 1.09–2.92, *P* = 0.020). Subgroup analysis showed that MELD-XI score still had prognostic value in the subgroup without chronic kidney disease (HR 3.30 95%CI 2.01–5.42 *P* < 0.001; HR_adjusted_ 1.88 95%CI 1.06–3.35 *P* = 0.032, *P* for interaction = 0.038).

**Conclusions:**

This study proved that the MELD-XI score at admission was related to the poor prognosis of hospitalized patients with chronic heart failure within 3 years.

## Introduction

Heart failure is a clinical syndrome that is the end-stage manifestation of a variety of cardiac diseases. Chronic heart failure aggravation is often accompanied by liver and kidney function damage; however, such damage is long-standing and further aggravated as heart failure progresses. The mechanism of hepatic and renal impairment is most likely related to the long-term hypoperfusion, congestion and inflammatory response caused by chronic heart failure (CHF) (1–4).

The Model for End-stage Liver Disease (MELD) score has been validated and widely used for risk assessment in patients with advanced liver disease (5, 6). The MELD-XI score was one of several modified MELD scores and originally developed to assess severity of illness in patients with end-stage liver disease receiving oral anticoagulants to reasonably assign priority to liver transplantation (7). Several studies have shown that the MELD-XI score was associated with the prognosis of a variety of cardiovascular diseases (8–10). Previous studies have confirmed that hepatic and renal impairment are common conditions in patients with acute heart failure and that mortality is highly positively correlated with the MELD-XI score (11, 12). However, there is currently less evidence for adverse outcomes in patients with chronic heart failure. Recently, it has been noted that the MELD-XI score predicts adverse events in the short term in patients with heart failure with preserved ejection fraction (HFpEF) (13). In addition, the association of the MELD score with the occurrence of adverse long-term outcomes was also observed in a study in an end-stage heart failure population who received cardiac resynchronization therapy (CRT) (14). However, there is currently a lack of evidence on the relationship of the MELD-XI score to long-term outcomes in the hospitalized CHF population.

## Methods and materials

### Data source and study definition

#### Study population

This was a single-center retrospective observational cohort study. A total of 465 patients who were first hospitalized due to chronic heart failure from February 2017 to December 2017 from the Department of Geriatrics, the Second Affiliated Hospital of Chongqing Medical University, were enrolled. Chronic heart failure was defined according to the diagnostic criteria established by 2021 ESC Guidelines for the diagnosis and treatment of acute and chronic heart failure (14): (1) Signs and symptoms of heart failure are present; (2) LVEF <50%, or LVEF ≥50% with evidence of cardiac structural and/or functional abnormalities consistent with left ventricular diastolic dysfunction/elevated left ventricular filling pressure.

Exclusion criteria: (1) Acute heart failure, acute coronary syndrome, and acute cerebral stroke; (2) Patients with malignancies, including solid tumors but also hematologic malignancies; (3) Existing severe hepatic and renal insufficiency (chronic kidney disease stage V and Child-C stage); (4) Missing data on serum creatinine or bilirubin within 24 h of admission.

In this study, liver and kidney dysfunction was defined as liver or renal impairment not caused by heart failure

#### Measurement of indicators of interest

Liver and kidney function markers were measured at the hospital testing center (Hitachi RL7600 Automatic Biochemical Analyzer). The upper limit of total bilirubin is 20.4 μmoL/L, and the upper limit of serum creatinine is132.3 μmoL/L.

The calculation formula of the MELD-XI score follows the setting of previous research: 5.11 × Ln [total bilirubin (mg/dl)] +11.76 × Ln [creatinine (mg/dl)] + 9.44 (15). The MELD-XI score >9.44 was considered elevated (12).

#### Follow-up and outcome

Outcome was defined as all-cause mortality within 3 years of admission. Follow-up of patients after discharge is by telephone, email, outpatient clinic, etc.

### Statistical analysis

Patients were divided into high MELD-XI group (≥9.44) and low MELD-XI group (<9.44) according to the MELD-XI score. There was a small proportion of missing values in the raw data, so imputation of missing values was performed by random forest interpolation using the MissForest package (16). The normal distribution of each continuous variable was assessed with the use of the Shapiro–Wilk test because of the small sample size. Nonnormally distributed continuous variables were presented as the median [25th−75th percentile]. Pearson's χ^2^ test and Fisher's exact test were used for categorical variables and the Mann-Whitney test was used for nonnormally distributed continuous variables. Variables with significant differences at baseline were further included in the univariate Cox regression analysis. Lasso regression was used to screen variables to build a predictive model. Decision curve analysis was used to assess the ability of the model to predict. Survival curves were estimated using the Kaplan–Meier method and compared by the log rank test. Statistical significance was set at *P* < 0.05 (two-sided). Data analysis was performed using IBM SPSS statistical software for Mac version 26.0 (Chicago, IL, USA) and R language (R for Mac version 4.2.0, R Foundation for Statistical Computing, Vienna).

## Results

### Characteristics of patients

Twenty-three patients were excluded due to tumors. Forty patients were excluded due to acute coronary syndrome. Two patients were excluded for lack of creatinine or bilirubin within 24 h of admission. A total of 400 patients were included in the final study. During the 3-year follow-up period, there were 97 all-cause deaths, including 63 cardiac deaths. The baseline information of the patients is shown in Table 1.

**Table 1:** Baseline characteristics of study population.

	**Overall population (*N* = 400)**	**Low MELD-XI (*N* = 256)**	**High MELD-XI (*N* = 144)**	***P*-value**
Age (year)	76.00 [67.00, 83.00]	75.00 [67.00, 81.00]	78.00 [66.00, 85.00]	0.075
Gender (female)	206 (51.50)	113 (44.14)	93 (64.58)	<0.001^*^
IBM, kg/m^2^	23.35 [21.08, 25.65]	23.28 [21.03, 25.69]	23.52 [21.09, 25.64]	0.970
SBP, mmHg	130.00 [118.00, 143.00]	129.00 [118.00, 142.00]	131.00 [118.25, 145.75]	0.611
DBP, mmHg	75.00 [67.00, 85.00]	75.00 [67.00, 83.00]	74.50 [66.00, 86.00]	0.796
HFrEF, *n* (%)	79 (19.75)	49 (19.14)	30 (20.83)	0.830
HFmrEF, *n* (%)	55 (13.75)	34 (13.28)	21 (14.58)	
HFpEF, *n* (%)	266 (66.50)	173 (67.58)	93 (64.58)	
NYHA II, *n* (%)	119 (29.75)	91 (35.55)	28 (19.44)	0.001^*^
NYHA III, *n* (%)	209 (52.25)	129 (50.39)	80 (55.56)	
NYHA IV, *n* (%)	72 (18.00)	36 (14.06)	36 (25.00)	
**Medical history**				
Hypertensive heart disease, *n*	77 (19.25)	50 (19.53)	27 (18.75)	0.849
Dilated cardiomyopathy, *n*	48 (12.00)	27 (10.55)	21 (14.58)	0.233
Rheumatic heart disease, *n*	32 (8.00)	22 (8.59)	10 (6.94)	0.006^*^
Coronary heart disease, *n*	233 (58.25)	149 (58.20)	84 (58.33)	0.287
Chronic kidney disease, *n*	74 (18.50)	9 (3.52)	65 (45.14)	<0.001^*^
Hepatopathy, *n*	26 (6.50)	12 (4.68)	14 (9.72)	0.058
COPD, *n*	54 (13.50)	34 (13.28)	20 (13.88)	0.864
Heart valve disease, *n*	162 (40.50)	94 (36.72)	68 (47.22)	0.040^*^
Arrhythmia, *n*	251 (62.75)	155 (60.55)	96 (66.67)	0.224
Hypertension, *n*	260 (65.00)	171 (66.80)	89 (61.80)	0.315
Diabetes mellitus, *n*	120 (30.00)	77 (30.08)	43 (29.86)	0.964
Smoking history, *n*	139 (34.75)	80 (31.25)	59 (40.97)	0.050
Drinking history, *n*	102 (25.50)	62 (24.22)	40 (27.78)	0.433
Previous PCI, *n*	91 (22.75)	58 (22.66)	33 (22.92)	0.952
Previous stroke /TIA, *n*	120 (30.00)	71 (27.73)	49 (34.03)	0.187
Atrial fibrillation, *n*	191 (47.75)	112 (43.75)	79 (54.86)	0.033^*^
PAD, *n*	298 (74.50)	191 (74.61)	107 (74.31)	0.947
**Echocardiogram**				
IVST, mm	11.00 [10.00, 12.00]	11.00 [10.00, 12.00]	11.00 [10.00, 12.00]	0.907
LAD, mm	42.00 [37.00, 48.00]	41.00 [37.00, 46.00]	45.00 [39.00, 50.00]	<0.001^*^
RATD, mm	40.00 [34.25, 45.00]	38.00 [34.00, 43.00]	42.00 [36.00, 48.75]	<0.001^*^
LVEDD, mm	50.00 [44.00, 58.00]	48.00 [42.25, 57.00]	53.00 [47.25, 63.00]	<0.001^*^
RVEDD, mm	24.00 [21.00, 27.00]	23.00 [21.00, 26.00]	25.00 [22.00, 28.00]	<0.001^*^
LVPWT, mm	10.00 [9.00, 11.00]	9.00 [8.00, 10.00]	10.00 [9.00, 11.00]	0.003^*^
LVEF, %	61.00 [43.00, 71.00]	63.00 [46.00, 72.00]	57.50 [39.00, 69.00]	0.007^*^
**Laboratory data**				
D-dimer, mg/L	0.10 [0.10, 0.10]	0.10 [0.10, 0.10]	0.10 [0.10, 0.28]	<0.001^*^
RBC, 10^12^/L	4.17 [3.75, 4.55]	4.19 [3.78, 4.53]	4.13 [3.67, 4.58]	0.519
Hb, g/L	125.00 [113.00, 138.00]	126.00 [115.00, 138.00]	124.00 [108.25, 138.00]	0.331
HCT	38.40 [34.30, 41.78]	38.60 [34.73, 41.48]	37.60 [33.30, 42.00]	0.263
MCV, fL	92.30 [89.30, 95.78]	92.10 [89.70, 95.50]	92.70 [88.03, 96.00]	0.994
MCH, pg	30.50 [29.30, 31.70]	30.70 [29.40, 31.70]	30.40 [28.58, 32.00]	0.452
MCHC, g/L	330.00 [320.00, 338.00]	330.00 [321.00, 338.75]	328.50 [317.25, 338.00]	0.256
RDW-CV	14.10 [13.23, 15.00]	13.80 [13.20, 14.70]	14.55 [13.70, 15.50]	<0.001^*^
RDW-SD, fl	46.00 [43.70, 49.40]	45.35 [43.20, 48.48]	47.70 [45.10, 51.88]	<0.001^*^
WBC, 10^9^/L	6.58 [5.16, 8.10]	6.50 [5.11, 7.96]	6.86 [5.24, 8.44]	0.247
Neutrophil, 10^9^/L	4.79 [3.57, 6.48]	4.64 [3.52, 6.31]	5.15 [3.77, 6.74]	0.036^*^
Lymphocyte, 10^9^/L	1.18 [0.88, 1.56]	1.23 [0.93, 1.58]	1.09 [0.74, 1.51]	0.002^*^
Monocyte, 10^9^/L	0.41 [0.28, 0.56]	0.41 [0.28, 0.52]	0.41 [0.29, 0.62]	0.166
Platelet, 10^9^/L	157.00 [124.25, 195.00]	160.00 [132.00, 201.50]	153.50 [111.25, 191.25]	0.008^*^
PTA, %	87.00 [72.00, 97.00]	91.00 [78.00, 100.00]	78.00 [63.00, 95.00]	<0.001^*^
INR	1.09 [1.02, 1.25]	1.06 [1.00, 1.17]	1.18 [1.04, 1.35]	<0.001^*^
PT, s	14.10 [13.30, 15.50]	13.80 [13.10, 14.90]	14.75 [13.50, 16.68]	<0.001^*^
APTT, s	36.95 [33.50, 40.50]	36.00 [33.30, 39.88]	38.20 [34.53, 42.78]	0.002^*^
Fibrinogen, g/L	3.36 [2.81, 4.33]	3.32 [2.88, 4.18]	3.46 [2.68, 4.56]	0.823
TT, s	17.30 [16.40, 18.40]	17.10 [16.30, 18.40]	17.60 [16.63, 18.60]	0.019^*^
K^+^, mmol/L	3.98 [3.71, 4.31]	3.95 [3.70, 4.24]	4.10 [3.74, 4.53]	0.026^*^
Na^+^, mmol/L	140.60 [137.73, 143.30]	140.95 [138.03, 143.78]	140.10 [137.00, 142.68]	0.043^*^
Scr, mg/L	0.94 [0.76, 1.23]	0.83 [0.69, 0.96]	1.37 [1.10, 1.82]	<0.001^*^
UA, μmol/L	411.15 [331.78, 518.00]	379.45 [306.18, 457.15]	507.00 [410.05, 604.28]	<0.001^*^
BUN, mmol/L	7.41 [5.86, 9.77]	6.65 [5.34, 8.09]	9.89 [7.49, 13.21]	<0.001^*^
lactic acid, mmol/L	2.30 [1.72, 3.00]	2.30 [1.70, 2.90]	2.40 [1.80, 3.14]	0.322
NTproBNP, pg/ml	2,116.50 [630.40, 5,550.50]	1,360.50 [438.60, 3,750.50]	4,243.50 [1,556.75, 9,995.50]	<0.001^*^
Albumin, g/L	37.20 [34.65, 39.80]	37.90 [35.20, 40.40]	36.15 [33.44, 39.08]	<0.001^*^
ALT, U/L	17.00 [11.00, 27.00]	18.00 [12.00, 28.00]	16.00 [11.00, 25.00]	0.252
AST, U/L	22.00 [17.00, 31.00]	22.00 [17.00, 29.75]	23.00 [17.00, 31.00]	0.432
ALP, U/L	75.00 [60.00, 93.00]	74.00 [59.00, 92.00]	77.50 [63.00, 93.00]	0.176
GGT, U/L	37.00 [23.00, 71.00]	34.00 [21.00, 68.75]	41.50 [25.25, 75.50]	0.069
TBIL, mg/dl	0.68 [0.50, 0.98]	0.62 [0.45, 0.81]	0.90 [0.62, 1.39]	<0.001^*^
TBA, μmol/L	4.50 [2.60, 8.10]	4.50 [2.50, 7.48]	4.85 [2.70, 8.60]	0.194
Prealbumin, mg/L	194.50 [143.00, 233.75]	201.50 [160.00, 237.50]	180.00 [124.25, 229.75]	0.004^*^
TG, mmol/L	1.07 [0.80, 1.44]	1.12 [0.84, 1.47]	0.98 [0.71, 1.38]	0.001^*^
TC, mmol/L	3.59 [2.97, 4.34]	3.73 [3.11, 4.43]	3.27 [2.69, 4.20]	<0.001^*^
HDL-C, mmol/L	1.12 [0.92, 1.34]	1.14 [0.98, 1.35]	1.02 [0.84, 1.31]	0.001^*^
LDL-C, mmol/L	1.90 [1.45, 2.39]	1.96 [1.55, 2.42]	1.71 [1.40, 2.29]	0.012^*^
ApoA1, g/L	1.35 [1.13, 1.57]	1.39 [1.22, 1.63]	1.28 [1.01, 1.45]	<0.001^*^
ApoB, g/L	0.80 [0.64, 0.96]	0.82 [0.69, 0.97]	0.74 [0.59, 0.95]	0.014^*^
ApoE, mg/L	34.20 [28.50, 39.40]	34.35 [28.83, 39.63]	33.85 [28.00, 38.78]	0.476
Lp (a), mg/L	143.20 [79.90, 234.10]	139.15 [75.20, 217.78]	153.90 [95.93, 242.85]	0.098
HbA1C, %	6.48 [6.20, 6.91]	6.47 [6.20, 6.95]	6.48 [6.23, 6.89]	0.808
MELD-XI	7.04 [4.29, 11.47]	5.25 [2.38, 6.69]	12.71 [10.88, 15.44]	<0.001^*^
**Medication use**				
β-blocker, *n* (%)	238 (59.50)	158 (61.72)	80 (55.56)	0.228
AECI, *n* (%)	120 (30.00)	71 (27.73)	49 (34.03)	0.187
ARB, *n* (%)	131 (32.75)	90 (35.15)	41 (28.47)	0.172
Aspirin, *n* (%)	104 (26.00)	73 (28.52)	31 (21.53)	0.126
Statin drug, *n* (%)	248 (62.00)	165 (64.45)	83 (57.64)	0.178
Clopidogrel, *n* (%)	189 (47.25)	126 (49.22)	63 (43.75)	0.293
NOAC, *n* (%)	38 (9.50)	28 (10.94)	10 (6.94)	0.191
Warfarin, *n* (%)	52 (13.00)	33 (12.89)	19 (13.19)	0.931
CCB, *n* (%)	123 (30.75)	78 (30.47)	45 (31.25)	0.871
Diuretic, *n* (%)	322 (80.50)	196 (76.56)	126 (87.50)	0.008^*^
Hypoglycemic agent, *n* (%)	98 (24.50)	65 (25.39)	33 (22.92)	0.581
ARNI, *n* (%)	17 (4.25)	10 (3.91)	7 (4.86)	0.650
Digoxin, *n* (%)	133 (33.25)	78 (30.46)	55 (38.19)	0.115

*Data are n/N (%) or median [25th−75th percentile]*.

* *P <0.05*.

*IVST, interventricular septal thickness; LAD, left atrial diameter; RATD, right atrial transverse diameter; LVEDD, left ventricular end diastolic diameter; RVEDD, right ventricular end diastolic diameter; LVPWT, late diastolic left ventricular posterior wall thickness; HCT, hematocrit; MCV, mean corpuscular volume; MCH, Mean RBC hemoglobin content; MCHC, mean corpuscular hemoglobin concentration; RDW-CV, coefficient of variation of red cell distribution width; RDW-SD, red cell distribution width standard deviation; PTA, plasma prothrombin activity; INR, International normalized ratio; APTT, activated partial thromboplastin time; PT, prothrombin time, TT, thrombin time; UA, uric acid; BUN, blood urea nitrogen; TBA, total bile acid; TBIL, total bilirubin; Scr, serum creatinine; TG, triglyceride; TC, total cholesterol; LDL-C, low density lipoprotein cholesterol; HDL-C, high density lipoprotein cholesterol; Apo, apolipoprotein; SBP, systolic pressure; DBP, diastolic blood pressure; NOAC, non-vitamin K antagonists oral anticoagulants; CCB, calcium channel blocker; ARNI, angiotensin receptor neprilysin inhibitor; PAD, peripheral arterial disease; PCI, percutaneous transluminal coronary Intervention; TIA, transient ischemic attack; ACEI, angiotensin-converting enzyme inhibitor; ARB, angiotensin receptor blocker*.

From the baseline table, the patients were older overall, but there was no difference between the two groups. In terms of medical history, the high MELD-XI score group had a higher prevalence of chronic kidney disease, valvular heart disease, rheumatic heart disease, and atrial fibrillation compared to the low MELD-XI score group. The high MELD-XI score group also had higher rates of diuretic use. The results of cardiac ultrasound indicated that the cardiac chambers of the patients in the high scoring group might be relatively large with a lower LVEF. There are many differences between the two groups of patients in terms of laboratory data. Compared to the low MELD-XI score group, the high MELD-XI score group had higher D-dimer, coefficient of variation of red cell distribution width (RDW-CV), red cell distribution width standard deviation (RDW-SD), Neutrophil, INR, prothrombin time (PT), activated partial thromboplastin time (APTT), thrombin time (TT), K^+^, serum creatinine (Scr), uric acid (UA), blood urea nitrogen (BUN), NT-proBNP and total bilirubin (TBIL) while Na^+^, lymphocyte, platelet, albumin, prealbumin, triglyceride (TG), total cholesterol (TC), high density lipoprotein cholesterol (HDL-C), low density lipoprotein cholesterol (LDL-C), apolipoprotein A1(ApoA1) and ApoB were lower.

### Lasso regression to screen variables

**Figure 1:**
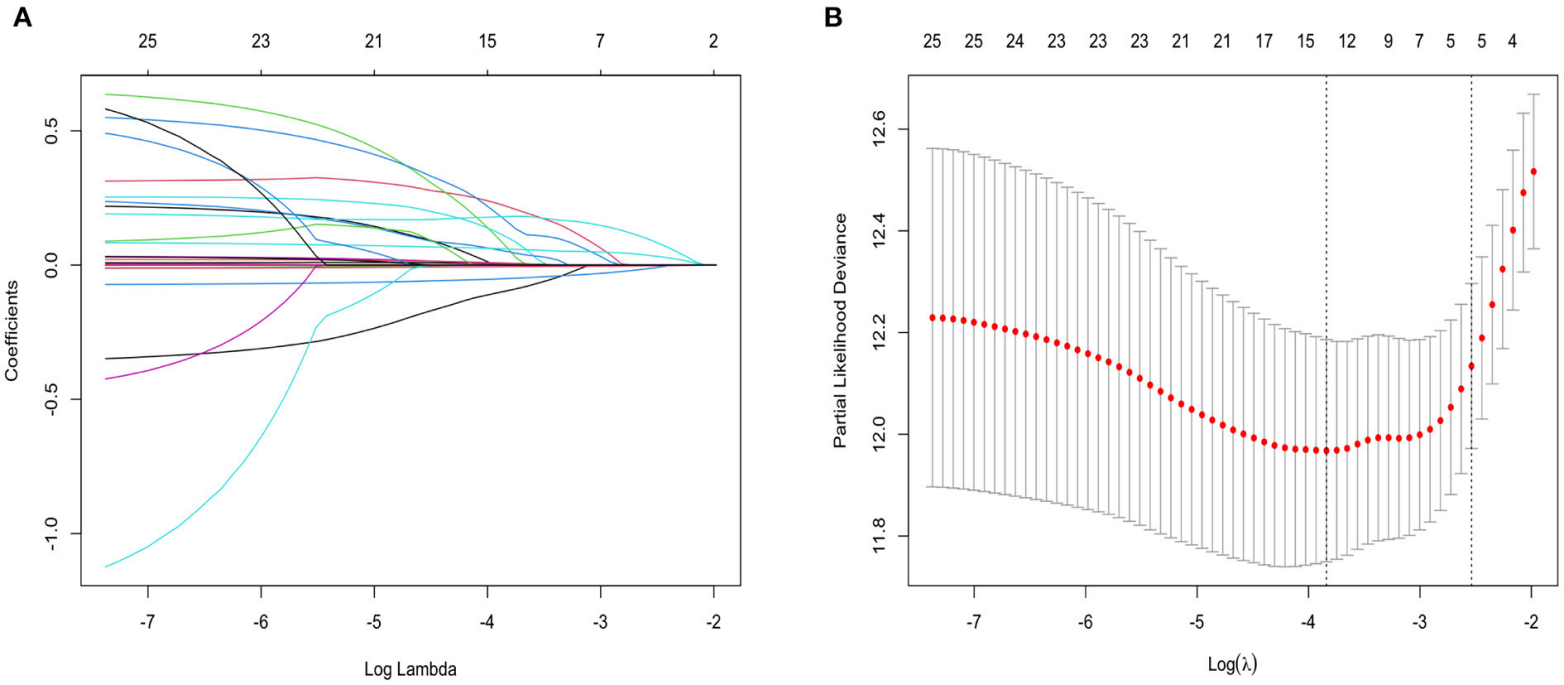
**(A)** Lasso regression fitting Cox regression model; **(B)** Tenfold cross validation. The curves in **(A)** represent each variable that enters lasso regression analysis. The two vertical dashed lines in **(B)** represent the minimum penalty value (λ value) and the penalty value at the first standard deviation respectively. In **(A)**, the point where the vertical line corresponding to the position of the penalty value intersects the curve is the number of filtered variables, and the ordinate of the corresponding intersection is the regression coefficient of the variable.

Variables with significant differences in the Table 1 were considered potential confounders and included in the univariate Cox regression analysis. The results of univariate Cox regression analysis are shown in Table 2. Variables with *P* < 0.1 in Table 2 were considered as confounders. However, since an excess of variables with significance was not suitable for using multivariate Cox regression, further variable screening was performed with lasso regression fitted with Cox equal proportional hazards models after removing blood creatinine and total bilirubin. After tenfold cross-validation, the model with the first standard error of the λ value was selected as the final model, which incorporated NT-proBNP, BUN, RDW-cv, Na^+^ and prealbumin, as shown in Figure 1.

**Table 2:** Results of univariate COX regression analysis.

	**HR**	**95%CI (Low)**	**95%CI (Up)**	***P*-value**
Gender(female)	1.23	0.82	1.84	0.318
NYHA II	1.00			
NYHA III	2.12	1.17	3.83	0.013^*^
NYHA IV	4.10	2.17	7.74	<0.001^*^
Rheumatic heart disease	0.81	0.35	1.85	0.615
Chronic kidney disease	2.43	1.59	3.73	<0.001^*^
Heart valve disease	1.28	0.86	1.92	0.23
Atrial Fibrillation	1.79	1.19	2.69	0.005^*^
LAD	1.01	0.99	1.03	0.413
RATD	1.05	1.03	1.07	<0.001^*^
LVEDD	1.02	1.00	1.04	0.057
RVEDD	1.06	1.02	1.10	0.003^*^
LVPWT	0.98	0.87	1.10	0.724
LVEF	0.99	0.98	1.00	0.056
D-dimer	2.06	1.36	3.12	0.001^*^
RDW-CV	1.38	1.24	1.55	<0.001^*^
RDW-SD	1.09	1.04	1.13	<0.001^*^
Neutrophil	1.01	0.98	1.03	0.707
Lymphocyte	0.55	0.37	0.84	0.005^*^
Platelet	1.00	0.99	1.00	0.238
PTA	0.99	0.98	1.00	0.022^*^
INR	1.27	0.79	2.06	0.329
PT	1.02	0.97	1.07	0.4
APTT	1.02	1.00	1.05	0.055
TT	1.00	0.98	1.02	0.696
K^+^	1.15	0.77	1.72	0.499
Na^+^	0.92	0.88	0.95	<0.001^*^
Scr	1.46	1.18	1.80	<0.001^*^
UA	1.00	1.00	1.00	0.365
BUN	1.11	1.07	1.15	<0.001^*^
NTproBNP	1.00	1.00	1.00	<0.001^*^
Albumin	0.90	0.85	0.94	<0.001^*^
TBIL	1.74	1.31	2.33	<0.001^*^
Prealbumin	0.99	0.99	0.99	<0.001
TG	0.71	0.48	1.06	0.091
TC	0.73	0.58	0.92	0.007^*^
HDL-C	9.46	1.00	0.00	0.002^*^
LDL-C	0.68	0.49	0.94	0.020^*^
ApoA1	0.28	0.15	0.53	<0.001^*^
ApoB	0.71	0.33	1.52	0.371
Diuretic	2.63	1.32	5.22	0.006^*^

* *P <0.05*.

*LAD, left atrial diameter; RATD, right atrial transverse diameter; LVEDD, left ventricular end diastolic diameter; RVEDD, right ventricular end diastolic diameter; LVPWT, late diastolic left ventricular posterior wall thickness; RDW-CV, coefficient of variation of red cell distribution width; RDW-SD, RBC distribution width standard deviation; PTA, plasma prothrombin activity; INR, International normalized ratio; APTT, activated partial thromboplastin time; PT, prothrombin time, TT, thrombin time; Scr, serum creatinine; UA, uric acid; BUN, blood urea nitrogen; TBIL, total bilirubin; TG, triglyceride; TC, total cholesterol; LDL-C, low density lipoprotein cholesterol; HDL-C, high density lipoprotein cholesterol*.

The MELD-XI score was incorporated into the newly established model. The situation of model improvement is shown in Table 3. Among them, the newly established model combined with the MELD-XI score has the lowest AIC value, so it is the optimal model.

**Table 3:** Comparison of different prognostic models on CHF patients.

**Model predictors**	**Likelihood ratio test χ^2^**	**AIC**	***P*-value**	**C-index**	**Adjusted C-index**
Model	81.81	995.19	<0.001	0.759	0.750
Model + Scr	82.47	996.53	<0.001	0.761	0.748
Model + TBIL	82.71	996.29	<0.001	0.759	0.748
Model + MEDEL-XI^*^	87.17	991.83	<0.001	0.764	0.751
Model + MEDEL-XI	84.55	994.44	<0.001	0.761	0.750

* *Hierarchical variable*.

*AIC, Akaike information criterion*.

**Figure 2:**
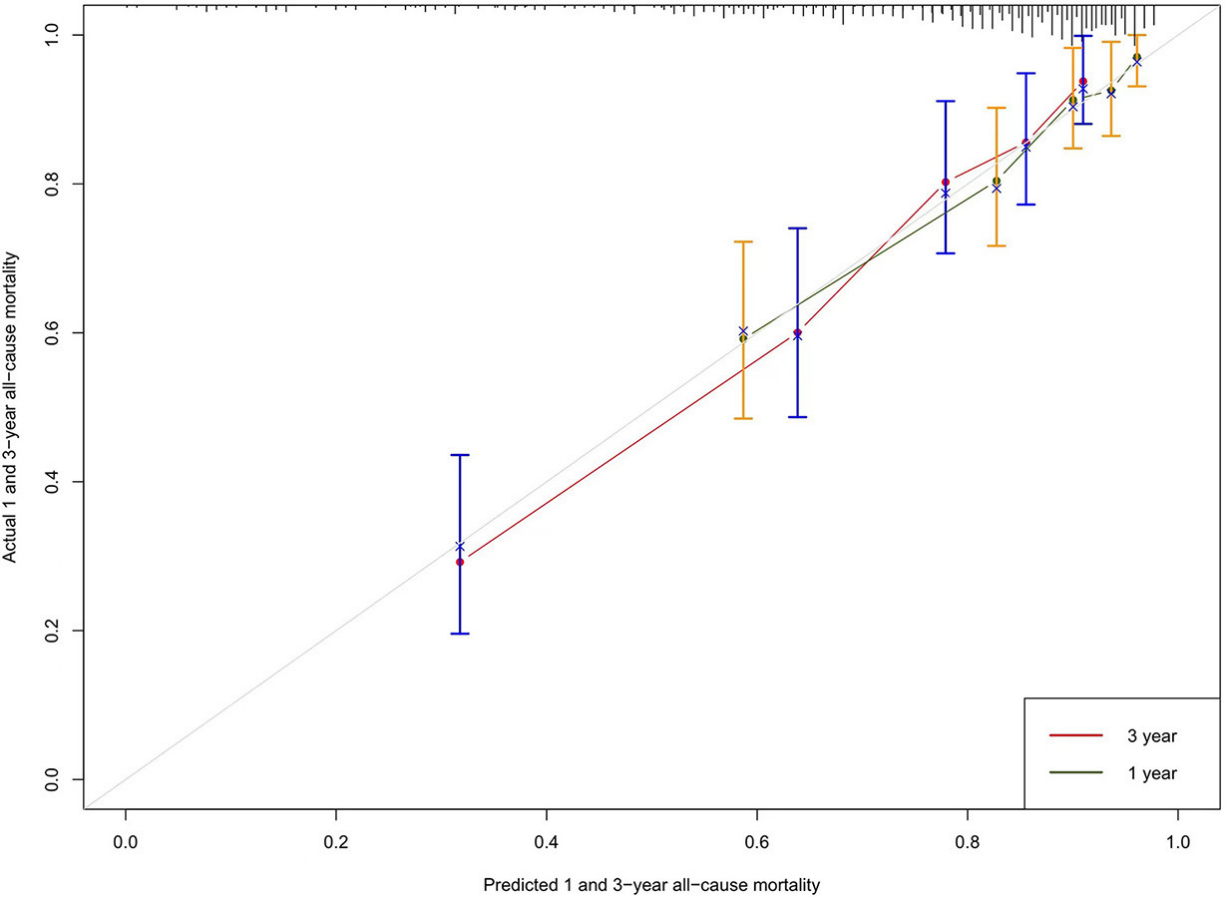
Calibration curve: The abscissa is the predicted event rate, and the ordinate is the observed actual event rate, both ranging from 0 to 1, which can be understood as the event rate (percentage). The gray line of the diagonal is the reference line, i.e. the case where the predicted value = the actual value. The gray green and red curves represent the 1 - and 3-year model fit lines, respectively, and the two sides with color parts are 95% confidence interval.

**Figure 3:**
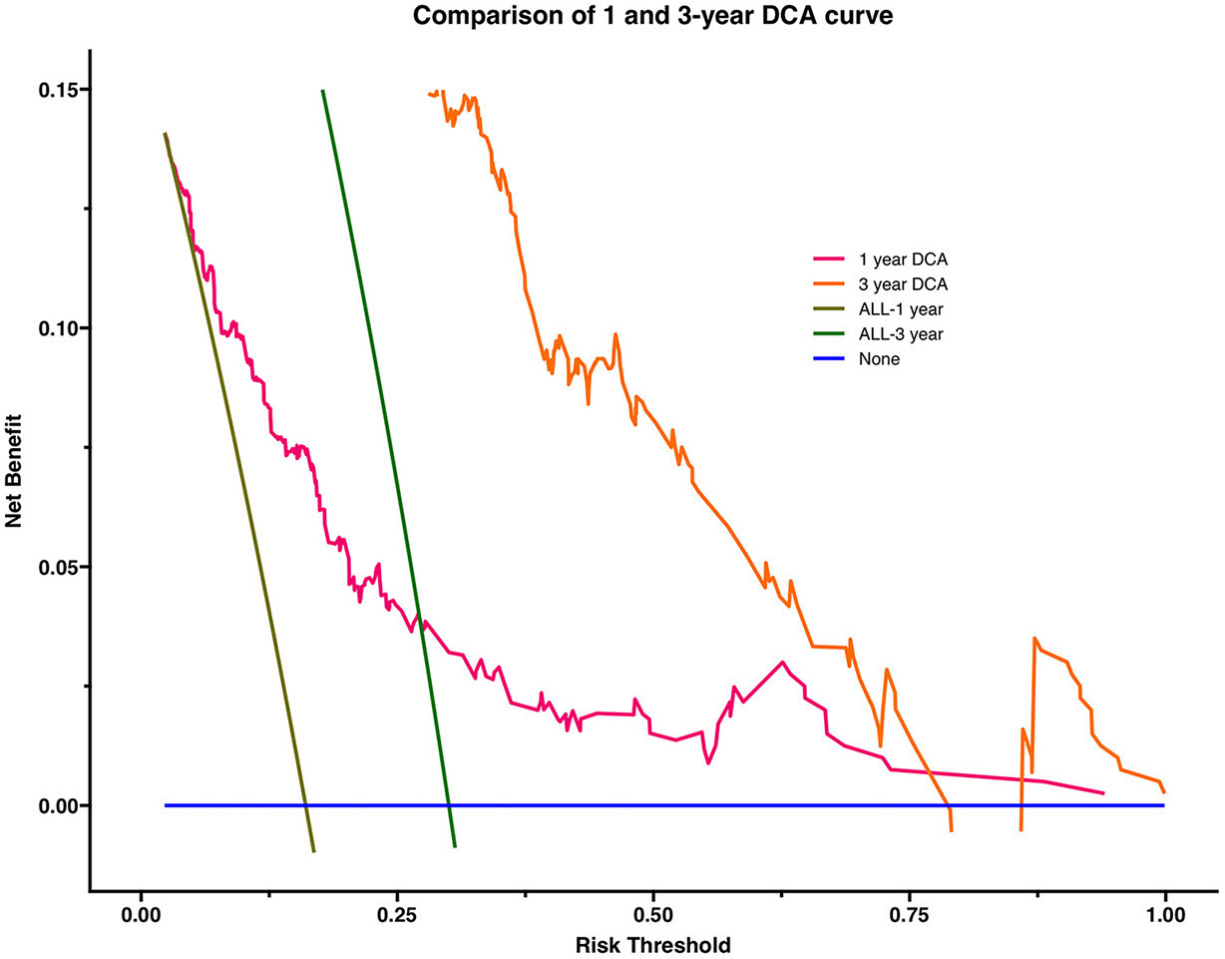
Comparison of 1 and 3-year DCA: The abscissa of the graph is threshold probability (threshold probability). All line and none line are representative of one extreme state. The all line represents all patients with a bad outcome and the none line represents all patients without a bad outcome. It can be seen from the figure that both the 1-year DCA curve and the 3-year DCA curve are above the extreme line, thus illustrating that the newly constructed prediction model is of clinical utility. The area under the curve was 0.030 for the 1-year DCA curve and 0.085 for the 3-year DCA curve, so there would be some improvement in a patient's risk of death within 3 years if interventions were required to be implemented using this model.

Calibration curves and decision curve analysis were used to evaluate the constructed model. The prediction analysis of 1- and 3-year all-cause mortality is presented in Figures 2, 3. The calibration curve of 1 and 3 years predicted by the model is close to the diagonal, indicating that a good accuracy of the model (Categorical variable MELD-XI + model) in predicting 1 and 3-year all-cause mortality (Figure 2). Furthermore, from the decision curve, although decision makers can use the model to have a net benefit in predicting all-cause mortality within 1 and 3 years in patients with CHF, the assessment of long-term prognosis can have a greater benefit (Figure 3).

### Survival analysis

**Figure 4:**
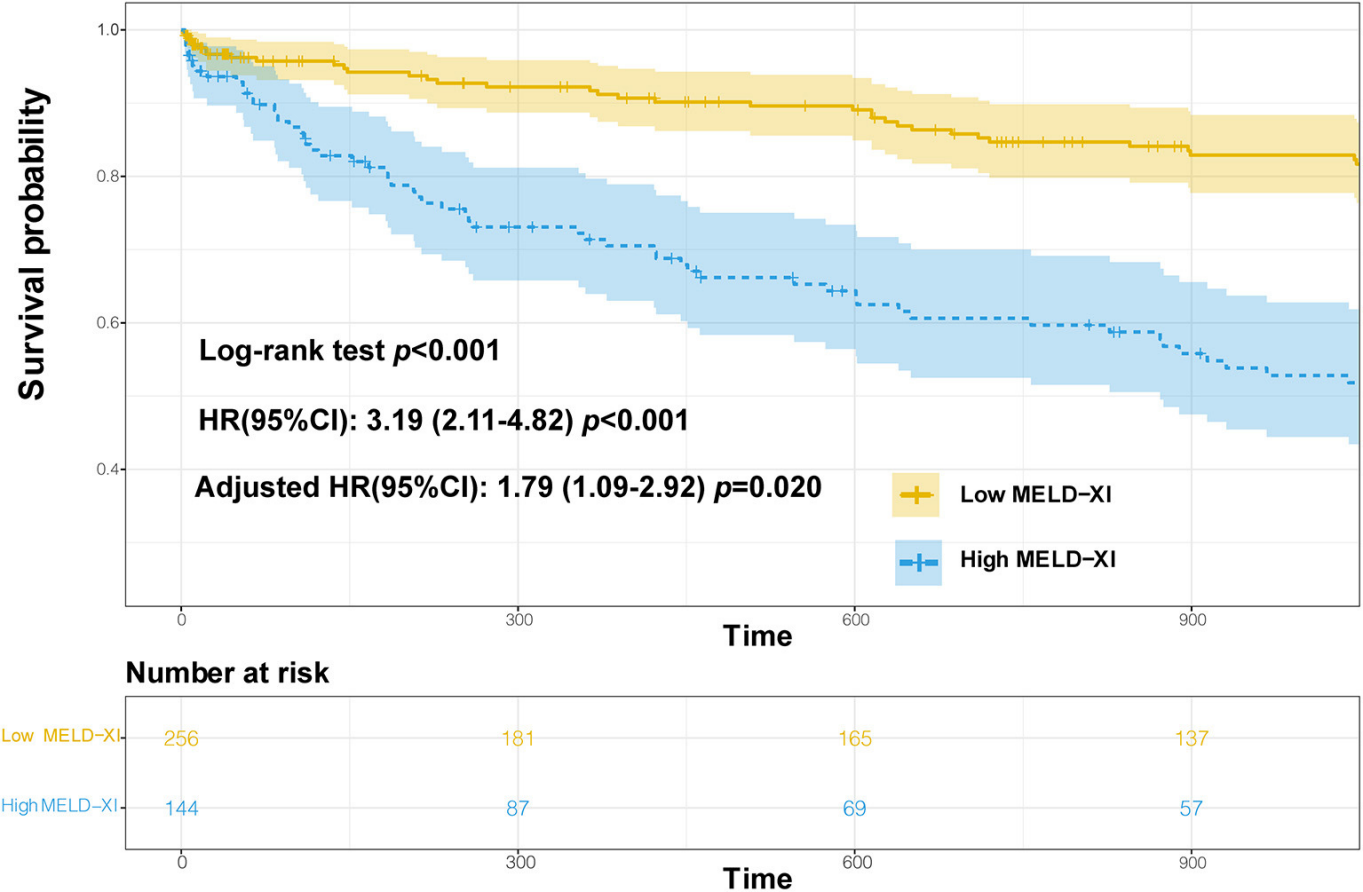
Survival curves.

The 3-year survival curve was plotted using the Kaplan–Meier method, and the results showed that the risk of all-cause mortality was significantly different between the two groups (Log-rank test *P* < 0.001),as shown in Figure 4. Cox regression analysis showed that the MELD-XI score was associated with the risk of all-cause mortality over 3 years in hospitalized CHF patients, and the risk of death in the high score group (MELD-XI score ≥9.44) was 3.19 times higher than that in the low score group (MELD-XI score <9.44; unadjusted HR 3.19, 95% CI 2.11–4.82, *P* < 0.001). After adjustment for NT-proBNP, BUN, RDW-cv, Na^+^ and prealbumin, the MELD-XI score still had some predictive value (HR 1.79, 95% CI 1.09–2.92, *P* = 0.020). For the results of the survival analysis, we calculated the post hoc power of the study (17). The structure shows that 99% power can explain the relationship between MELD-XI score and 3-year mortality in patients with CHF.

### Subgroup analysis

The association between high MELD-XI score (≥9.44) and the composite outcome in subgroups is shown in Table 4. There was an interaction effect in the subgroups with and without CKD, but no significant interaction effect was observed among the other subgroups. The MELD-XI score had significant prognostic value in patients without CKD subgroup (HR 3.30 95%CI 2.01–5.42 *P* < 0.001; HR_adjusted_ 1.88 95%CI 1.06–3.35 *P* = 0.032). This illustrates that the MELD-XI score may be more suitable for prognostic evaluation in patients without CKD.

**Table 4:** The subgroup analyses of MELD-XI score and 3-year all-cause mortality of heart failure.

**Group**	**High MELD-XI^†^**	**Low MELD-XI^†^**	**HR (95%CI)**	***P*-value**	***P* for interaction**	**HR_adjusted_ (95%CI)**	$P_{\text{adjusted}}^{*}$	***P* for interaction**
All	58/144	39/256	3.19 (2.11–4.82)	<0.001		1.79 (1.09–2.91)	0.020	
**Age**					0.286		
<65	6/51	7/28	3.60 (2.30–5.63)	<0.001		0.38 (0.09–2.47)	0.193	0.184
≥65	51/116	33/205	1.80 (0.60–5.35)	0.294		2.17 (1.28–3.67)	0.004	
**Gender**					0.286		
Male	33/93	13/113	4.11 (2.07–8.13)	<0.001		2.33 (1.05–5.14)	0.037	0.967
Female	25/51	26/143	3.20 (1.85–5.56)	<0.001		1.58 (0.82–3.06)	0.175	
**Types of heart failure**					0.218		
HFrEF	10/30	9/49	1.66 (0.68–4.09)	0.269		1.26 (0.43–3.69)	0.668	0.534
HFmrEF	8/21	5/34	2.59 (0.85–7.94)	0.095		1.52 (0.43–5.34)	0.514	
HFpEF	40/93	25/173	4.12 (2.46–6.89)	<0.001		1.59 (0.82–3.09)	0.174	
**NYHA grade**					0.966		
II	6/28	8/99	2.46 (0.85–7.11)	0.095		0.42 (0.07–2.56)	0.346	0.868
III	31/80	22/129	2.95 (1.68–5.17)	<0.001		1.91 (1.00–3.64)	0.051	
IV	21/36	9/36	2.66 (1.21–5.83)	0.015		2.01 (0.79–5.10)	0.141	
**Chronic kidney disease**					0.049		
Yes	28/65	4/9	1.04 (0.37–2.97)	0.941		0.68 (0.22–2.15)	0.516	0.038
No	30/79	35/247	3.30 (2.01–5.42)	<0.001		1.88 (1.06–3.35)	0.032	
**Hepatopathy**					0.726		
Yes	8/14	3/12	2.20 (0.58–8.35)	0.248		1.36 (0.17–10.04)	0.791	0.229
No	50/130	36/244	3.15 (2.04–4.87)	<0.001		1.65 (0.98–2.77)	0.059	
**Heart valve disease**					0.669		
Yes	29/68	17/94	3.53 (1.89–6.60)	<0.001		1.98 (0.95–4.16)	0.070	0.908
No	29/76	22/162	2.89 (1.66–5.04)	<0.001		1.39 (0.72–2.71)	0.330	
**Atrial fibrillation**					0.901		
Yes	22/65	22/122	2.92 (1.69–5.05)	<0.001		2.62 (1.37–5.03)	0.004	0.796
No	22/65	17/144	3.21 (1.70–6.05)	<0.001		0.97 (0.40–2.35)	0.951	
**Hypertension**					0.530		
Yes	35/89	25/171	3.55 (2.10–6.02)	<0.001		1.73 (0.88–3.41)	0.115	0.373
No	23/55	14/85	2.62 (1.35–5.09)	0.005		1.75 (0.81–3.80)	0.158	
**Diabetes mellitus**					0.097		
Yes	15/43	14/77	1.96 (0.95–4.07)	0.07		0.85 (0.33–2.34)	0.747	0.121
No	43/101	25/179	3.94 (2.37–6.55)	<0.001		2.19 (1.21–3.96)	0.010	
**Smoking history**					0.529		
Yes	22/59	11/80	4.09 (1.88–8.91)	<0.001		2.44 (0.90–6.62)	0.081	0.956
No	36/256	28/176	2.94 (1.87–4.81)	<0.001		1.75 (0.99–3.11)	0.056	

*CI, confidence interval; HR, hazard ratio*.

*Covariates used for adjustment: DBP, RDW-CV, BUN, NT-proBNP, Prealbumin, Na^+^.

† *Death events/ patients*.

## Discussion

This study proves applicability of the MELD-XI score in hospitalized patients with CHF and reveal that the MELD-XI score can be used to assess long-term prognosis in this population. Liver and kidney function impairment is a common complication in patients with heart failure, meanwhile, many studies have also confirmed that relevant indicators of liver and kidney function are also powerful predictors in patients with heart failure (18–20). Decompensation of cardiac function caused hypoperfusion of the liver and kidney, which led to decreased glomerular filtration rate, in addition to increased pressure in the right heart that further aggravated systemic circulation congestion, which further aggravated hepatic ischemia and hypoxia. This is currently a common explanation for the mechanism of liver and kidney impairment due to heart failure, but it is important to note that many patients with CHF can achieve liver and kidney function improvement after effective medical therapy, and for such patients, whether the MELD-XI score continues to be applicable remains to be investigated.

This study considered the association between baseline MELD-XI score and mortality in hospitalized patients with CHF and did not explore outcomes in patients with improved or worsened MELD-XI score. What we need to understand is that CHF is persistent and continues to worsen, and so-called improvement in heart failure as mere improvement in symptoms. Similarly, if patients have persistent chronic heart failure, hepatic and renal impairment cannot be truly resolved, and improvement in liver and renal function measures may simply reflect symptomatic improvement. Previous studies have noted that not every creatinine rise has a deleterious effect, and it may be a marker that relief of congestion is associated with better outcomes (21). Furthermore, In addition, although there is insufficient evidence that renal damage occurs earlier than liver damage, elevated bilirubin may be a major contributor to the elevated MELD-XI score and its prognosis (12), and also implies cardiac decompensation and possibly the need for aggressive inotropic agents (22). Therefore, in terms of the MELD-XI score at baseline, we boldly speculate that MELD-XI score not only reflects the liver and kidney function damage, but also reflects the organ function reserve status of patients with heart failure. The elevation of MELD-XI score suggests that during the progression of chronic heart failure, these patients have poor hepatic and renal functional reserves and cannot adapt to long-term chronic injury, making them more likely to experience adverse outcomes early. The MELD-XI score sheds some light on the fact that for the management of patients with chronic heart failure, it is not enough to relieve symptoms alone, and how to improve the prognosis is also a matter of great consideration. Improving hepatic and renal reserve may lead to better outcomes for patients with heart failure, which can be achieved by treating the primary disease, reducing the use of drugs with hepatic and renal impairment, and rational medication, etc.

In the final step of the study, we performed a post hoc subgroup analysis, so its results need to be interpreted with caution. The results show that there are interaction effects between subgroups with or without chronic kidney disease, which indicates that MELD-XI score is likely to be suitable for patients with chronic heart failure without chronic kidney disease. We agree with this point because if a patient presents with primary kidney disease, their serum creatinine is likely to be consistently high, which is likely to introduce bias into the study.

There are some limitations of our study that cannot be ignored. First, the problems with observational studies themselves are difficult to avoid, and although we used lasso regression for screening variables, there are some potential confounders that cannot be avoided. Second, the overall population in the study was older, and its applicability is open to debate. Finally, although we calculated the power post hoc for the overall population, the results of subgroup analysis of subgroup analysis were worrisome due to the small sample size, so we highly recommend extending the sample size of the subgroups for further confirmation.

## Conclusions

An elevated MELD-XI score on admission most likely implies poor hepatic and renal functional reserve in patients with hospitalized chronic heart failure and is associated with poor long-term outcomes.

## Data availability statement

The original contributions presented in the study are included in the article/supplementary material, further inquiries can be directed to the corresponding author.

## Ethics statement

Ethical review and approval was not required for the study on human participants in accordance with the local legislation and institutional requirements. Written informed consent for participation was not required for this study in accordance with the national legislation and the institutional requirements.

## Author contributions

ZL proposed the idea of the study. ZL and XL performed the writing of the article and data analysis. LX, YL, CQ, and SS were involved in data collection and patient screening. YZ and LZ reviewed the entire study as well as the final manuscript for revision. All authors contributed to the article and approved the submitted version.

## Conflict of interest

The authors declare that the research was conducted in the absence of any commercial or financial relationships that could be construed as a potential conflict of interest.

## Publisher's note

All claims expressed in this article are solely those of the authors and do not necessarily represent those of their affiliated organizations, or those of the publisher, the editors and the reviewers. Any product that may be evaluated in this article, or claim that may be made by its manufacturer, is not guaranteed or endorsed by the publisher.
